# Role of an elliptical structure in photosynthetic energy transfer: Collaboration between quantum entanglement and thermal fluctuation

**DOI:** 10.1038/srep26058

**Published:** 2016-05-13

**Authors:** Hisaki Oka

**Affiliations:** 1 Institute for Research Promotion, Niigata University, 8050, Ikarashi 2-no-cho, Nishi-ku, Niigata 950-2101, Japan

## Abstract

Recent experiments have revealed that the light-harvesting complex 1 (LH1) in purple photosynthetic bacteria has an elliptical structure. Generally, symmetry lowering in a structure leads to a decrease in quantum effects (quantum coherence and entanglement), which have recently been considered to play a role in photosynthetic energy transfer, and hence, elliptical structure seems to work against efficient photosynthetic energy transfer. Here we analyse the effect of an elliptical structure on energy transfer in a purple photosynthetic bacterium and reveal that the elliptical distortion rather enhances energy transfer from peripheral LH2 to LH1 at room temperature. Numerical results show that quantum entanglement between LH1 and LH2 is formed over a wider range of high energy levels than would have been the case with circular LH1. Light energy absorbed by LH2 is thermally pumped via thermal fluctuation and is effectively transferred to LH1 through the entangled states at room temperature rather than at low temperature. This result indicates the possibility that photosynthetic systems adopt an elliptical structure to effectively utilise both quantum entanglement and thermal fluctuation at physiological temperature.

Quantum entanglement is a unique phenomenon in quantum systems. When two (or many) interacting systems are in the form of an inseparable state, which is a superposition state possessing quantum correlation, the system is said to be “entangled”. A well-known example of quantum entanglement is entanglement between photons, and various applications using the photon entanglement, such as quantum information technology (QIT)^1^,^2^,^3^, quantum metrology^4^, quantum microscopy^5^ and quantum control^6^,^7^, have been proposed. Recently, quantum entanglement in nanomaterials has also been investigated, especially in the field of QIT^8^,^9^,^10^,^11^. Generally, however, the creation of quantum entanglement in materials is not as easy as in photons because a strong coupling of quantum states at low temperature is required to avoid their decoherence due to thermal noise. Therefore, it was considered that quantum entanglement could not be formed in complex many-body systems at room temperature. Surprisingly, however, it has been recently predicted that quantum entanglement exists in biological systems^12^,^13^ at physiological temperature, especially in the light-harvesting complexes (LH) of a photosynthetic system^12^.

A number of theoretical and experimental studies have already suggested that quantum coherence exists in photosynthetic systems in the form of an exciton state^14^,^15^,^16^,^17^,^18^. In fact, LH has a highly symmetric structure, which facilitates the delocalisation of excitons formed therein. In particular, purple photosynthetic bacteria, which is a species of photosynthetic bacteria, have two types of LH, namely, LH1 (core antenna) and LH2 (peripheral antenna); it has been revealed that LH2 has a circular structure with almost complete rotational symmetry^19^. However, high-resolution X-ray experiments have revealed that LH1 in purple photosynthetic bacteria has an elliptical structure rather than circular one^20^,^21^,^22^,^23^. In addition, some of these experiments also have suggested that a gap exists in the elliptical structure^20^,^21^,^22^. Generally, symmetry lowering in structure and structural distortion lead to spatial anisotropy and localisation of a wave. Therefore, an elliptical structure seems to work against efficient energy transfer utilising the wavelike exciton states. Though the optical properties of the elliptical LH1 have been extensively investigated, the effect of elliptical distortion on the energy transfer from LH2 to LH1 remains unknown.

Here we theoretically analyse in detail the effect of elliptical distortion on photosynthetic energy transfer in terms of quantum entanglement. We also clarify how a gap in the elliptical structure influences the energy transfer process. From numerical results, it is shown that in the presence of elliptical distortion, quantum entangled states between LH1 and LH2 are formed at higher energy levels than they would be if LH1 had a circular structure. In other words, this indicates that elliptical distortion creates efficient energy transfer pathways at high energy levels. At room temperature, light energy absorbed by LH2 is thermally pumped with the help of thermal fluctuation and is transferred to LH1 through the entangled states. Consequently, though quantum coherence is sacrificed, both the time scale of energy transfer and the amount of transfer energy are improved at room temperature rather than at low temperature. This result indicates the possibility that photosynthetic systems adopt an elliptical structure to effectively utilise both quantum entanglement and thermal fluctuation at physiological temperature. Meanwhile, a gap in the elliptical structure leads to large decrease in the amount of transfer energy, whereas the time scale of energy transfer shortens slightly. From this result, the gap have little benefit, at least, for the energy transfer from LH2 to LH1.

## Results

### Effect of elliptical distortion on quantum entanglement between LH1 and LH2

LH in photosynthetic bacteria consists of carotenoid and bacteriochlorophyll (BChl), which harvest photons of different wavelengths. In particular, BChl is referred to as B800, B850 and B875, named after its absorption wavelength. For purple photosynthetic bacteria, energy transfer pathways between the BChls have been fully elucidated: B800 → B800 (within LH2), B800 → B850 (within LH2), B850 → B850 (within LH2), B850 → B875 (LH2 → LH1) and LH1 → reaction centre (RC)^24^. In addition, there are two classes of RC-LH1, i.e. monomeric and dimeric; the monomeric consists of one RC surrounded by one LH1, whereas the dimeric consists of two RC-LH1s. In this study, we focus on a monomeric RC-LH1 in *Rhodopseudomonas acidophila*, which is one of the most intensively studied purple photosynthetic bacteria, and restrict ourselves to analysing the B850 → B875 (LH2 → LH1) pathway.

**Figure 1:**
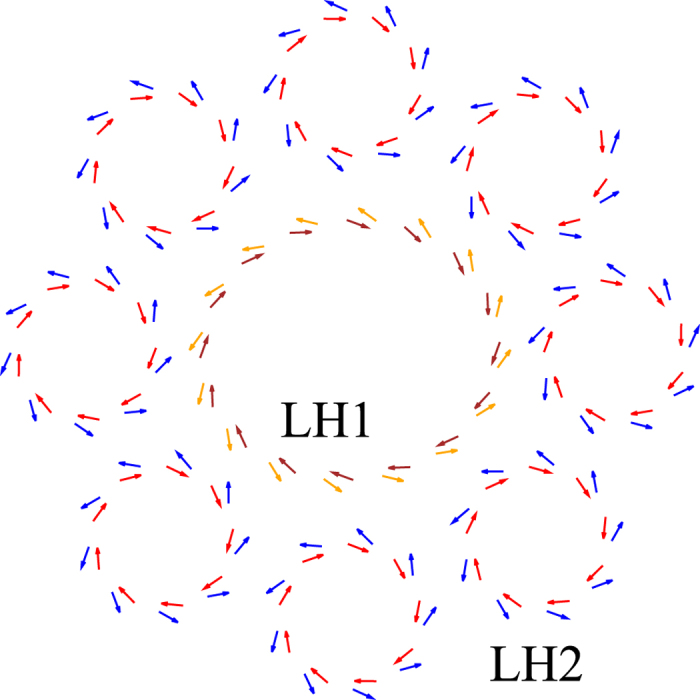
Schematic of the model. The arrows indicate the dipole moments of BChls.

An analytical model is depicted in Fig. 1, where BChls are represented as colour-coded arrows indicating their dipole moments. LH1 consists of 32 elliptically distributed B875s, where the long and short axes of the ellipse are set to 11 nm and 9.5 nm^20^, respectively. LH2 consists of 18 concentrically arranged B850s, and 8 LH2s (144 B850s in total) surround the LH1 in such a way that the shortest distance between neighbouring LH2s becomes ~2 nm^25^,^26^ and the total energy of the whole system becomes minimal.

Generally, the interaction energy and energy difference between BChls strongly influence the time scale and efficiency of energy transfer. These influences can be quantified by the degree of quantum entanglement between the donor and acceptor^27^; rapid and efficient energy transfer occurs via strongly entangled states formed between the two. Therefore, in order to clarify how an elliptical structure influences the energy transfer process, we analyse the effect of elliptical distortion on the formation of quantum entanglement between LH1 and LH2. Though there are some measures of quantum entanglement, namely, relative entropy of entanglement^28^ and entanglement of formation^29^, a rigorous method for evaluating the degree of entanglement for arbitrary many-body systems has not been established. However, the degree of entanglement for arbitrary bipartite many-level systems can be rigorously evaluated using the relative entropy of entanglement. As shown below, the system considered here can be reduced to a bipartite many-level system by introducing exciton bases, and we can adopt the relative entropy of entanglement.

First, we start by formulating the quantum states of BChls and the interaction between them. Near its absorption wavelength, each BChl can be approximated by a two-level system with a ground state |*g*〉 and an excited state |*e*〉, where |·〉 is the Dirac ket vector indicating a quantum state. By numbering all BChls in LH1 and LH2s (hereinafter the site representation), an excited state can be denoted as 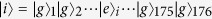, which indicates that only the *i*th BChl is excited and the others are in the ground state. The interaction between BChls can be approximated by dipole-dipole interaction. Defining the relative distance vector between the *i*th and *j*th BChls as **r**_*ij*_ ≡ **r**_*i*_ − **r**_*j*_, the dipole-dipole interaction is given by *V*_*ij*_ = *κ*_*ij*_/4*πϵ*|**r**_*ij*_|^3^ with *κ*_*ij*_ = **d**_*i*_ · **d**_*j*_ − 3(**d**_*i*_ · **r**_*ij*_)(**d**_*j*_ · **r**_*ij*_)/|**r**_*ij*_|^2^, where **d**_*i*_ is the dipole moment of the *i*th BChl and *ϵ* is the local dielectric constant. Using the rotational-wave approximation, the interaction Hamiltonian in the site representation can be described as


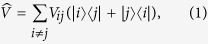


where the sum for *i* and *j* is taken for all BChls in LH1 and LH2.

The above site representation is adequate for analysing the energy transfer between BChls. For strongly interacting BChls, however, the use of eigenstates (exciton states) significantly simplifies the analysis as shown below. The exciton state is defined as a superposition of quantum states in the form of the site representation. By considering LH1 and LH2 separately, the exciton states can be described formally as 
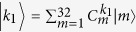
 for LH1 and 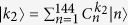 for LH2, where *k* is the quantum number of the exciton state and *C*^*k*^ is the expansion coefficient. Using the exciton bases of |*k*_1_〉 and |*k*_2_〉, equation (1) can be rewritten as


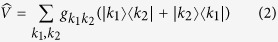


and the total Hamiltonian can then be described as 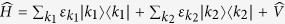, where *ε*_*k*_ is the exciton energy and 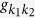 is the coupling energy of excitons. Thus, by introducing the exciton bases, the many-body problem of energy transfer between BChls can be reduced to the analysis of energy transfer in a simple bipartite many-level system, i.e. {|*k*_1_〉} and {|*k*_2_〉}, and hence we can apply the relative entropy of entanglement directly to this system.

**Figure 2:**
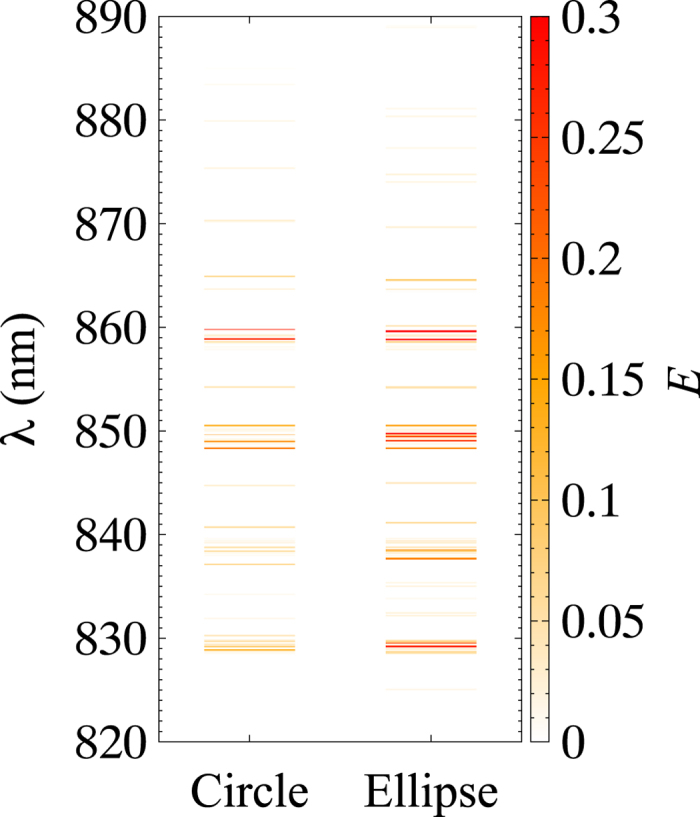
Relative entropies of entanglement, *E*, for two types of LH1: Circle and elliptical LH1s.

We can now evaluate the degree of entanglement, *E*, between LH1 and LH2. In Fig. 2, *E* is shown as a colour map on the corresponding exciton levels. For the parameters of B850, we referred to ref. 30, and for LH1, we presume that B875 has the same dipole moment as B850 because the detailed structure of LH1 has not been fully elucidated. For comparison, we first calculate *E* for a circular LH1 with a radius of 5.1 nm (‘Circle’ column in Fig. 2). One can see that strong entanglement is formed intensively at exciton levels near 860 nm: *E* = 0.277 (maximum) at *λ* = 859.7 nm and *E* = 0.263 at *λ* = 858.9 nm. These levels correspond to optically-allowed exciton states in LH2. The maximum except for the above two levels is *E* = 0.187 at *λ* = 848.3 nm. From this result, in the circular LH1, both photon absorption and energy transfer occur mainly near 860 nm. In contrast, for the elliptical LH1 (Ellipse), strong quantum entanglements are formed widely on the short-wavelength side compared with the circular LH1: *E* = 0.299 (maximum) at *λ* = 859.6 nm, *E* = 0.288 at *λ* = 858.8 nm, *E* = 0.284 at *λ* = 849.7 nm, *E* = 0.23 at *λ* = 849.3 nm, *E* = 0.245 at *λ* = 849 nm, *E* = 0.234 at *λ* = 829.5 nm and *E* = 0.277 at *λ* = 829.2 nm. This indicates that elliptical LH1 forms efficient transfer pathways on the short-wavelength side. Thus, elliptical distortion leads to a major difference in the formation of quantum entanglement and creates strong entanglements at high energy levels.

### Entanglement dynamics in energy transfer process

Next, we analyse the dynamics of energy transfer at two temperatures (77 K and 300 K) and clarify how the difference in the formation of quantum entanglement affects the time scale and efficiency of energy transfer. Though some methods of evaluating the time scale of energy transfer, such as Förster theory^31^, extended Förster theory^32^,^33^ and modified Redfield theory^34^, are known, they are not suitable for the analysis of quantum entanglement because in these theories, interactions between BChls are treated as perturbations or the total Hamiltonian of BChls is diagonalised as a whole. In this study, we therefore adopt standard quantum master equation^35^ in which only the effects of dumping due to the environment of BChls are treated as perturbations.

Using the natural units of *ħ* = 1, the quantum master equation in the interaction picture is given by


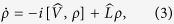


where *ρ* is the density operator describing the quantum states of LH1 and LH2, and 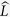 denotes the superoperator describing the dumping effects due to the environment. Since the time scale of spontaneous emission of BChl is ~1 ns and is about 1000 times as long as that of energy transfer, the effect of spontaneous emission in 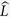 can be ignored. In addition, in order to focus on how the quantum entanglement between LH1 and LH2 affects the energy transfer process, we ignore the effect of strong correlation with the environment, such as non-Markovian process. Consequently, we take into account only the effects of thermal relaxation and dephasing due to the environment in 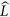. For the detailed theoretical description of 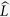 can be found in Methods section; a standard thermal bath model consisting of oscillators in thermal equilibrium at temperature *T* is adopted, in which *T* is introduced via the mean phonon number, 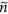, so that the effect of thermal pumping due to thermal fluctuation can be properly included.

**Figure 3:**
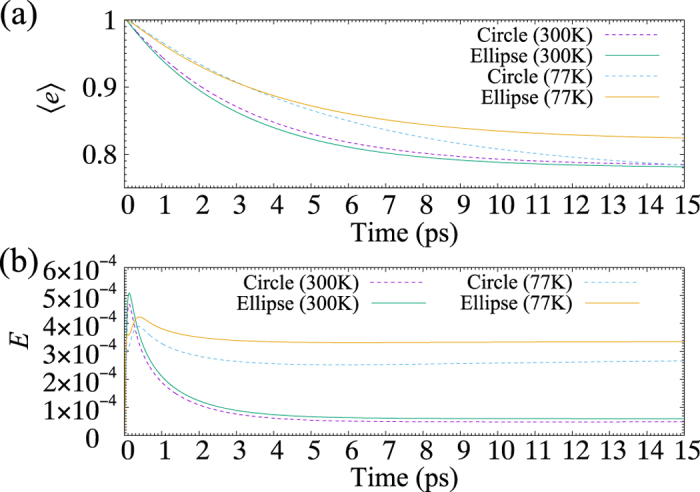
(**a**) 〈*e*〉 as a function of time, where 〈*e*〉 is the total population of excited states of LH2. (**b**) *E* as a function of time.

Figure 3 shows 〈*e*〉 and *E* as functions of time, where 〈*e*〉 is the total population of excited states of LH2 and the change in 〈*e*〉 corresponds to the amount of transfer energy. Supposing optical excitation, we assume that the population of the exciton state with the largest dipole moment in LH2 is initially 1. The parameters of BChls are the same as those in Fig. 2. As the result in Fig. 3(a) shows, both the time scale of energy transfer and the amount of transfer energy at 300 K are improved by elliptical distortion. The time scale of energy transfer is ~3.3 ps for Circle (dashed line) and ~3 ps for Ellipse (solid line); elliptical distortion shortens the time scale by 10%. In the amount of transfer energy, Ellipse surpasses Circle. In contrast, at 77 K, the presence of elliptical distortion considerably decreases the amount of transfer energy. The corresponding dynamics of *E* are shown in Fig. 3(b). Owing to decoherence effect, the LH1-LH2 system forms a mixed state, for which we cannot directly evaluate *E* of individual LH1-LH2 coupled excitons as in Fig. 2. Hence, *E* in Fig. 3(b) indicates the whole quantum entanglement formed between LH1 and LH2. Since 176 exciton states form a mixed state, the value of *E* becomes significantly small compared with *E* in Fig. 2. At 300 K, *E* reaches its peak at ~0.2 ps and decreases with time. On the other hand, at 77 K, *E* reaches its peak at ~0.4 ps and decreases with time, and then increases very gradually after ~7 ps. Intriguingly, *E* reaches its maximum at 300 K rather than at 77 K in spite of a strong decoherence effect, and elliptical distortion increases *E* at both 77 K and 300 K. Thus, elliptical distortion improves both the time scale of energy transfer and the amount of transfer energy at 300 K. This result indicates that the quantum entangled states formed at high energy levels effectively contribute to energy transfer only at 300 K.

**Figure 4:**
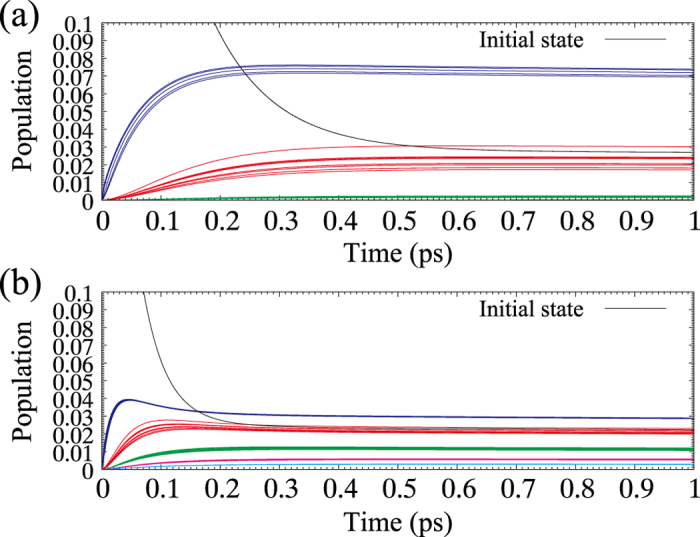
Population dynamics of individual LH2 excitons: (**a**) at 77 K and (**b**) at 300 K.

The mechanism of the efficient energy transfer due to the elliptical structure can be explained by verifying the population dynamics of individual exciton states of LH2, in which we can see that the thermal pumping plays an important role. Figure 4 shows population dynamics of individual LH2 excitons at 77 K and 300 K. The exciton states are colour-coded according to wavelength: the lowest energy band near 864 nm (blue line), the second lowest energy band near 860 nm (red line), the middle energy band near 850 nm (green line), the second highest energy band near 840 nm (pink line) and the highest energy band near 830 nm (sky-blue line) (see Numerical calculation in Methods section for details). Calculation parameters are the same as those in Fig. 3, in which the population of optically-allowed exciton near 860 nm is initially 1 (black line). Owing to the thermal relaxation, the population of initial states gradually decreases and then other exciton states are excited by thermal pumping. At 77 K, the thermal energy is about 6.6 meV, corresponding to a wavelength difference of Δ*λ* ≈ 3.9 nm, and the lowest-energy and the second lowest-energy bands (red and blue) are mainly excited. Since the exciton states near the lowest-energy exciton band have little quantum entanglement, the exciton states only near 860 nm contribute to the energy transfer process. At room temperature (300 K), however, the thermal energy is about 26 meV on average, corresponding to Δ*λ* ≈ 14 nm, which is enough to pump up to higher-energy exciton bands. In fact, as can be seen in Fig. 4, exciton states up to the highest-energy band (sky blue) are strongly excited and hence, for elliptical structure, the quantum entanglement formed over a wider range of exciton levels can contribute to the energy transfer process. Thus, though the quantum coherence is sacrificed, the efficient energy transfer can be achieved with the help of the quantum entanglements formed over a wide range of exciton levels and the thermal fluctuation at room temperature.

### Effect of a gap in the elliptical structure

**Figure 5:**
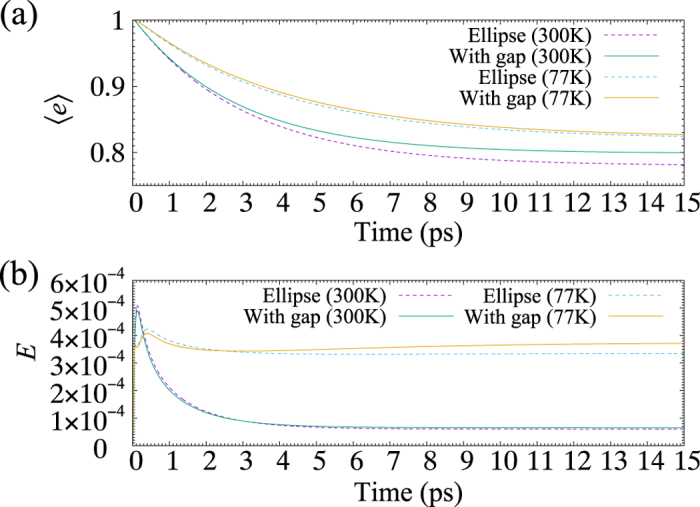
(**a**) 〈*e*〉 as a function of time. 〈*e*〉 is the total population of excited states of LH2. (**b**) *E* as a function of time. In (**a**,**b**), 〈*e*〉 and *E* for ‘Ellipse’ in Fig. 3 are replotted for comparison.

Finally, in this section, we analyse the effect of a gap in the elliptical structure on energy transfer. Figure 5 shows 〈*e*〉 and *E* as functions of time. For comparison, 〈*e*〉 and *E* for ‘Ellipse’ in Fig. 3 are replotted. In the calculation, with reference to ref. 21, a gap is introduced by removing a pair of B875s near the short axis of ellipse. As the result in Fig. 5(a) shows, the time scale of energy transfer in the presence of a gap is ~2.85 ps; the gap shortens the time scale by 5%. However, at 300 K, the amount of transfer energy is considerably decreased by the presence of a gap. On the other hand, at 77 K, no significant improvements can be found in both the time scale and the amount of transfer energy. Figure 5(b) shows the corresponding dynamics of *E*. Both at 300 K and 77 K, the presence of a gap slightly lowers *E* within the time scale of energy transfer (<3 ps), and then *E* increases with time after 3 ps; in particular at 77 K, *E* recovers as large as its maximum of *E*. However, this recovery of *E* after 3 ps has no influence on the time scale and the amount of transfer energy at both 77 K and 300 K, as can be seen in Fig. 5(a). Therefore, we conclude that the recovery of *E* originates from quantum entanglements which contribute little to energy transfer from LH2 to LH1.

## Summary and Discussion

In summary, we have theoretically investigated the effect of the elliptical structure of LH1 on the energy transfer from LH2 and LH1 in a photosynthetic purple bacterium, and have shown that the elliptical distortion improves both the time scale of energy transfer and the amount of transfer energy at room temperature. From the numerical calculation, it has been shown that by the presence of elliptical distortion, the quantum entangled states between LH1 and LH2 are formed over a wider range of high energy levels than would have been the case with circular LH1. At room temperature, light energy absorbed by LH2 is thermally pumped with the help of thermal fluctuation, and effectively transferred to LH1 through the entangled states formed at the high energy levels. As a consequence, though quantum coherence is sacrificed, both the time scale of energy transfer and the amount of transfer energy are improved at room temperature rather than at low temperature. This result indicates the possibility that photosynthetic systems adopt an elliptical structure to effectively utilise both quantum entanglement and thermal fluctuation at physiological temperature. Meanwhile, a gap in the elliptical structure leads to a large decrease in the amount of transfer energy, whereas the time scale of energy transfer slightly shortens. In addition, at both 300 K and 77 K, the presence of a gap increase and recover the quantum entanglement in the time range after ~3 ps, which corresponds to the time scale of energy transfer. From this result, the gap has little benefit, at least, for the energy transfer from LH2 to LH1. In light of the time scale of ~30 ps in the energy transfer from LH1 to RC, the gap might contribute to the LH1-RC energy transfer process.

In actual purple photosynthetic bacteria, it might be difficult to directly measure quantum entanglement because this measurement requires specific techniques, such as time-coincident counting^36^ of quantum-state tomography^37^. However, we could indirectly observe the effect of quantum entanglement on energy transfer via the enhancement of the energy transfer rate. According to recent theoretical studies, the entangled photons can be used for a new type of spectroscopy and population control of vibronic states^6^,^7^. Therefore, it might be possible to increase quantum entanglement in a photosynthetic system by irradiation of frequency-entangled photons^38^. Meanwhile, there are some theoretical studies advocating that photosynthetic systems are not entangled^39^,^40^. Seen from this point of view, experimental measurement of quantum entanglement is therefore important. In addition, in this study, we ignore the influence of correlation due to the interaction with the environment, for simplicity, in order to focus on the effect of elliptical structure of LH1. Though this might modify the obtained results, the details of the correlation are poorly understood. It is the next step to introduce the bath model in our calculation and to analyse how the obtained results are modified by the bath influence. Furthermore, recently, studies to emulate the energy transfer process in LH using artificial nanostructures have been evolved, and various next-generation technologies utilising quantum entanglement, such as energy teleportation^41^, quantum optical networks integrated in nanoscale devices^42^ and quantum simulation^43^,^44^,^45^, have been extensively investigated. The results obtained in this study can be applied directly to such device applications utilising quantum entanglement.

Finally, we mention the effect of elliptical distortion in LH2 on the energy transfer process. Until recently, the elliptical distortion in LH2 had also been investigated^46^,^47^,^48^,^49^,^50^. To the best of our knowledge, the ‘structural’ elliptical distortion has not been observed experimentally, but it has been settled that the ‘energetic’ elliptical distortion occurs in LH2. This is validated in the fact that theoretical analyses based on the energetic distortion well reproduce the experimental results of single molecule spectroscopy of LH2 at extremely-low temperatures (1.2 K ~ 4.2 K). In this study, however, we ignore the elliptical distortion in LH2 because the phonon relaxation and dephasing effects are considered at 77 K and 300 K in our calculation. According to our results, elliptical distortion effectively functions on the donor side. Therefore, we guess that this energetic elliptical distortion would contribute to the energy transfer from B800 to B850 in LH2.

## Methods

### Quantum entanglement measure

Quantum entanglement can be evaluated using the entropy of entanglement. When the density operator *ρ* is in a completely pure state, the entropy of entanglement uniquely determines the degree of entanglement of *ρ*. However, for the mixed states considered in this study, there are some measures, namely, relative entropy of entanglement^28^ and entanglement of formation^29^. In this study, the relative entropy of entanglement is used, defined as *E* = −Tr[*ρ* log_*d*_ *σ*] + Tr[*ρ* log_*d*_ *ρ*], where *d* is the dimension of *ρ* and *σ* is a density operator having no quantum entanglement. *E* = 0 denotes no entanglement and *E* = 1 denotes maximal entanglement. Generally, we have to properly choose *σ* such that *σ* satisfies min *D*(*ρ*||*σ*), where *D* denotes any measure of distance between *ρ* and *σ*. For the system considered in this study, *σ* is easily obtained by setting the off-diagonal parts of *ρ* associated with the interaction between LH1 and LH2 to zero, i.e. 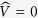.

### Numerical calculation

In an actual calculation, equation (3) is converted into a c-number equation by using the eigenstates of |*k*_1_〉 and |*k*_2_〉, e.g., as 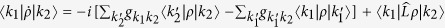, where 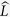 is the superoperator describing the effect of thermal relaxation and pure dephasing due to the environment, separated into two terms as 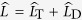. By defining the exciton states of LH1 and LH2 with the lowest energies, respectively, as 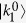 and 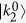, the thermal relaxation term, 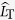, can be described as 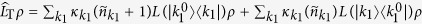  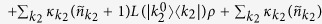 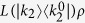 where *κ*_*k*_ is the relaxation rate due to phonons, 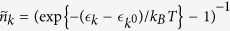 is the mean phonon number of the thermal bath at temperature *T*, obeying Planck’s law, and 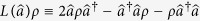 is the Lindblad-type superoperator. On the other hand, the pure dephasing term is described as 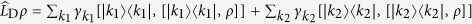, where *γ*_*k*_ is the dephasing rate due to the environment. We numerically solve the obtained c-number equation using the fourth-order Runge-Kutta method.

**Figure 6:**
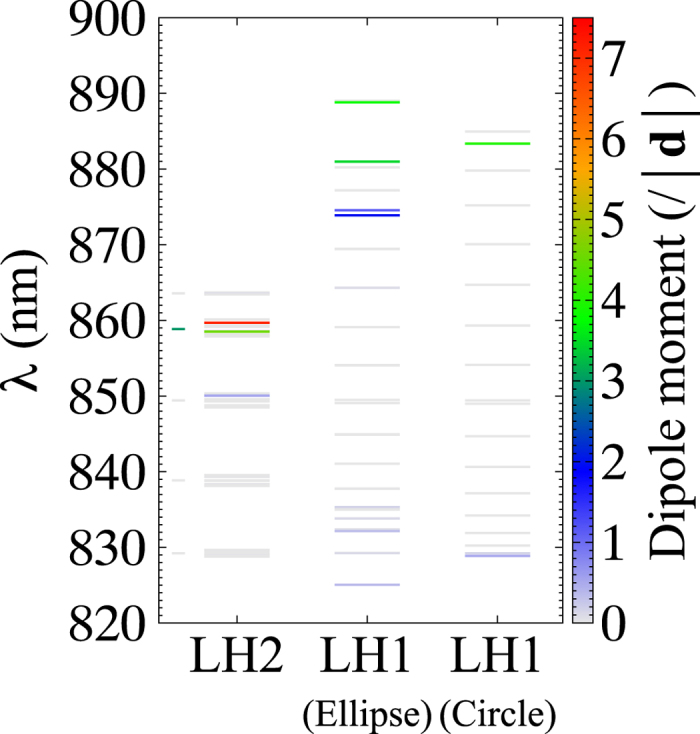
Example of the exciton levels of LH1 and LH2. The colour bar indicates the normalised dipole moment of the exciton state.

For the parameters, we use 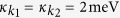 and 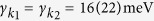 at *T* = 77 K (300 K), for simplicity. In addition, for BChls, we assume that the excitation energies of the *α*-bond and *β*-bond BChls in the site representation are identical, and we set them to 1.455 eV for LH1 and 1.5 eV for LH2 in such a way that the resonance levels of the excitons agree with experimental results^51^. The dipole moments of the BChls are also assumed to be identical, given by |**d**| = 6.5 Debye, and for the direction of the dipole moment, we referred to ref. 30, as mentioned in the text. The local dielectric constant is set to *ϵ* = 1.7. The resulting exciton levels are shown in Fig. 6. LH1 (Ellipse) is the exciton levels for the elliptical LH1 in Fig. 1. For comparison, the exciton levels for a circular LH1 with a radius of 5.1 nm is also given by the LH1 (Circle). For LH2, only the exciton band of the long-wavelength side is shown, and the exciton levels for a single LH2 are depicted by short solid lines besides the LH2 for reference. As is well known, most exciton states in LH2 and LH1(Circle) are degenerate. The colour map plotted on the energy levels indicates the dipole moment of exciton states normalised by that of a single BChl. For LH1 and LH2, most exciton states are optically forbidden. Strongly optically allowed exciton levels are found near 860 nm for LH2 and 880–890 nm for LH1.

## Additional Information

**How to cite this article**: Oka, H. Role of an elliptical structure in photosynthetic energy transfer: Collaboration between quantum entanglement and thermal fluctuation. *Sci. Rep.* **6**, 26058; doi: 10.1038/srep26058 (2016).
